# QuAPPro: an R shiny app for quantification and alignment of polysome profiles

**DOI:** 10.1186/s12859-026-06379-2

**Published:** 2026-01-22

**Authors:** Chiara Schiller, Matthias Lemmer, Sonja Reitter, Janina A. Lehmann, Kai Fenzl, Johanna Schott

**Affiliations:** 1 Mannheim Institute for Innate Immunoscience (MI3) and Mannheim Cancer Center (MCC), Medical Faculty Mannheim, Heidelberg University, Franz-Volhard-Str. 6, 68167 Mannheim, Germany; 2 Center for Molecular Biology of Heidelberg University (ZMBH) and German Cancer Research Center (DKFZ), DKFZ-ZMBH Alliance, Im Neuenheimer Feld 329, 69120 Heidelberg, Germany; 3 Institute for Computational Biomedicine, Faculty of Medicine, Heidelberg University Hospital and Heidelberg University, Im Neuenheimer Feld 130, 69120 Heidelberg, Germany; 4 Translational Spatial Profiling Center (TSPC), Heidelberg University Hospital, Heidelberg, Germany; 5 Present Address: Molecular Biology Laboratory (EMBL), Genome Biology Unit, Meyerhofstrasse 1, 69117 Heidelberg, Germany

**Keywords:** Translation, Protein biosynthesis, Polysomes, Polysome profiling, R shiny

## Abstract

**Background:**

Polysome profiling is a widespread technique to study mRNA translation. After separation of cellular particles by ultracentrifugation on a sucrose-density gradient, a UV absorbance profile is recorded during elution, which mostly reflects RNA content and shows distinct peaks for ribosomal subunits, monosomes and polysomes with increasing number of ribosomes. This profile can be used to assess global translational activity, or to reveal changes in ribosome biogenesis and translation elongation. In addition, it is also possible to measure the association of fluorescently tagged proteins with ribosomal subunits or polysomes. Alignment and quantification of polysome profiles usually relies on spreadsheet programs, custom R/Python scripts or commercial software.

**Results:**

With QuAPPro, we present the first interactive web app that allows quantification and alignment of polysome profiles, independently of the device or software that was used to generate the profiles. QuAPPro was written in R, with a graphical user interface implemented in R shiny. It supports interactive visualization and analysis of polysome profiles, including profile smoothing, baseline selection, alignment along a defined point on the x-axis, quantification of profile subsections and deconvolution for resolving individual peaks. Fluorescence profiles can be aligned and quantified in parallel. Finally, quantification results can be summarized and visualized as bar plots. Every interactive plot can be exported directly in a publication-ready format.

**Conclusions:**

This user-friendly tool does not only speed up the analysis of polysome profiles but also facilitates reproducibility and documentation of the process, without the need for programming abilities or commercial software.

**Supplementary Information:**

The online version contains supplementary material available at 10.1186/s12859-026-06379-2.

## Background

**Fig. 1:**
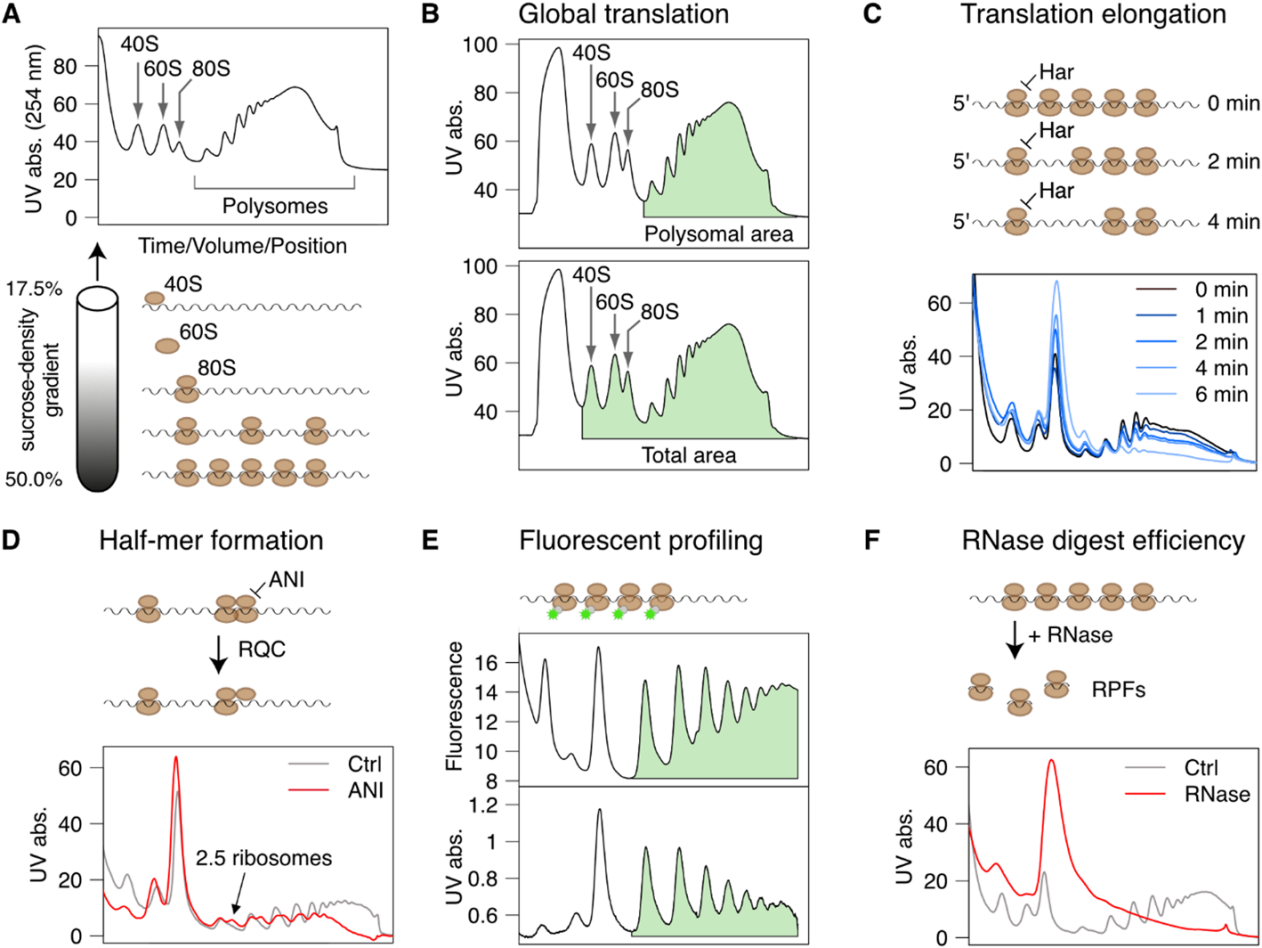
Polysome profiling application examples.** A** For polysome profiling, ribosomal subunits, monosomes and polysomes are separated by sucrose-density gradient ultracentrifugation. During elution, UV absorbance is recorded. **B** Assessment of the proportion of polysomal ribosomes (polysomal area/total area) as a proxy for global protein biosynthesis in RAW264.7 cells. **C** Measurement of translation elongation speed by ribosome run-off after stalling of initiating ribosomes with harringtonine (Har) in RAW264.7 cells. **D** Formation of half-mers as an intermediate step during ribosome quality control (RQC) to resolve ribosome collisions induced by the elongation inhibitor anisomycin (ANI). **E** Fluorescence and UV absorbance profile of yeast cells expressing GFP-tagged Ssb protein, which interacts with polysomes to assist nascent peptide folding. **F** Assessment of RNase A/T1 digest efficiency in RAW264.7 cells to generate ribosome protected fragments (RPFs) for ribosome profiling

After the first polysome profiles were published more than 60 years ago [1, 2], polysome profiling developed into an indispensable tool in the field of translation research. This technique can be used to study many different aspects of protein biosynthesis, such as global and mRNA-specific translation efficiency, ribosome biogenesis, ribosome quality control (RQC), co-translational nascent chain assembly as well as the association of regulatory factors with the translation machinery. Polysome profiling is based on the separation of ribosomal subunits, monosomes and polysomes by sucrose-density gradient ultracentrifugation. During elution of the gradient, UV absorbance at ~ 260 nm is recorded, which mostly reflects RNA content, except for the large peak at the beginning of the profile caused by detergents in the lysis buffer (Fig. S1). Further peaks represent small and large ribosomal subunits (40S and 60S in eukaryotes), monosomes and polysomes consisting of increasing numbers of ribosomes (Fig. 1A). As a common measure for global translation activity, the surface under the polysomal area of the profile (mRNAs with two or more ribosomes) serves as a proxy for the number of ribosomes that are engaged in active translation (Fig. 1B). In addition, the speed of translation elongation can be assessed in run-off assays where the initiating ribosome is stalled by harringtonine [3, 4] (Fig. 1C). Defects in ribosome biogenesis specifically affect the 40–60S peaks, and ribosomal subunit imbalance can lead to the appearance of additional peaks, so called half-mers. They represent stalled initiating 40S subunits waiting for 60S joining [5], or intermediate products during clearance of collided ribosomes [6] (Fig. 1D). The ribosome is a large protein complex that interacts with numerous regulatory factors such as initiation or elongation factors and chaperones that assist protein folding. Fluorescent polysome profiling allows the parallel recording of UV absorbance and the distribution of fluorescently tagged proteins [7]. Figure 1E shows a fluorescence profile of yeast cells expressing GFP-tagged Ssb protein, the yeast homologue of the chaperone Hsp70, which assists in protein folding of nascent peptides [8]. Moreover, polysome profiles are used to assess experimental manipulations such as limited enzymatic digest for ribosome profiling experiments [9–11] (Fig. 1F), and to study co-translational nascent chain interactions [12] or polysome assembly in vitro [13, 14]. As an alternative to sucrose-density gradient ultracentrifugation, size exclusion chromatography followed by uHPLC has been established, which leads to similar profiles [15].

So far, the analysis of polysome profiles is usually performed using spreadsheet programs like Excel, which is time-consuming, or custom scripts written in R or Python, which requires programming skills. While some devices used for recording UV absorbance offer software with options for alignment or quantification of profiles, advanced features like deconvolution of profiles into individual peaks usually rely on commercial software.

With QuAPPro, we present the first interactive web app that allows rapid and convenient quantitative analysis of polysome profiles. The app allows interactive alignment and facilitates both quantification of profile subsections and individual peaks by deconvolution. The analysis is independent of the device or software that was used to record the profiles. In addition, corresponding fluorescence profiles can be aligned and quantified for fluorescent polysome profiling.

## Implementation

QuAPPro was written in R [16], with a graphical user interface (GUI) implemented in R shiny [17]. We provide a server version with a manual, example data and a guided tour (https://www.umm.uni-heidelberg.de/mi3/biochemie/tools/quappro). The source code is openly available at our GitHub repository (https://github.com/johannaschott/QuAPPro).

The GUI focuses on interactive plots to make polysome profile analysis user-friendly and intuitive. The user imports one or more polysome profiles that are directly visualized. By clicking into the interactive plots, the user can set baselines, select anchors for alignment or define areas and peaks for quantification. Helper functions in the background ensure reproducibility. All intermediate plots as well as quantification tables are displayed and can be downloaded at any point of the analysis. In the following, we will describe the specific features and their implementation in more detail.

**Fig. 2:**
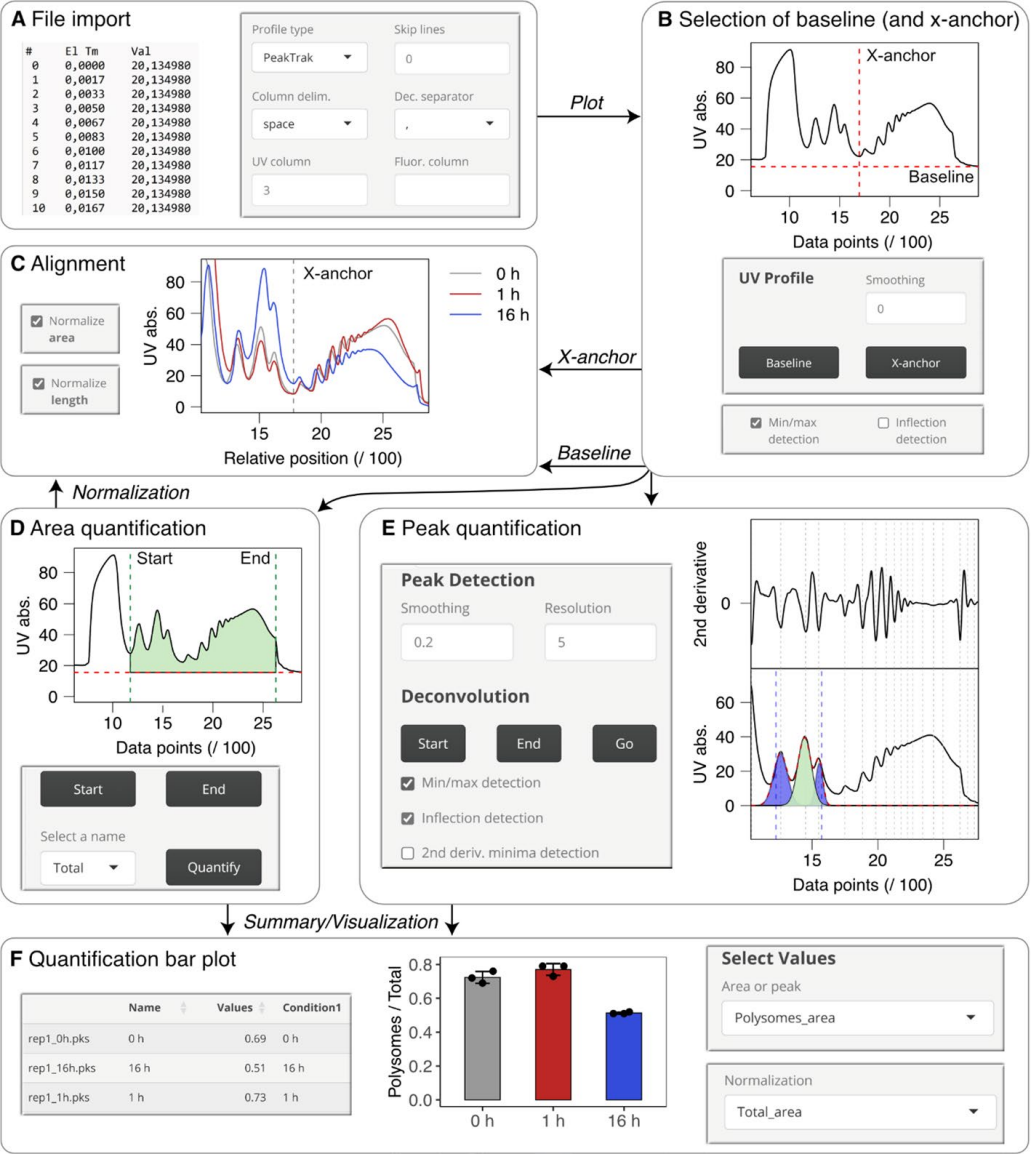
Overview of the QuAPPro analysis workflow.** A** Import of different file formats. **B** Interactive selection of baseline and x-anchor. **C** Alignment of multiple profiles with optional normalization for the total area and length of the profiles. **D** Quantification of profile subsections. **E** User-guided deconvolution and quantification of individual peaks. **F** Summary of quantification results and visualization as a bar plot

As input, text files containing a single profile per file (and if applicable the corresponding fluorescence profile) can be loaded. Several common input file formats exported from the PeakTrak (Teledyne Isco), TRIAX (BioComp) or PrimeView (Äkta) software can be selected (Fig. 2A). The respective settings are listed in Table S1. If none of the listed software outputs is used, the user can specify the column delimiter and decimal separator, a number of lines to be skipped from the beginning of the file, and the column number of the UV and possibly the fluorescence profile. The import parameters do not only depend on the software that generated the input file. The decimal separator is often determined by the language settings of the respective operating system. When a CSV (i.e. comma separated values) file is exported, also the column delimiter is affected by the language settings, because a semicolon is required when the comma is already used as a decimal separator. Therefore, also for files exported from the PeakTrak, TRIAX or PrimeView software, some settings may have to be adjusted by the user. If necessary, smoothing of UV absorbance or fluorescence profiles is possible (Fig. S2).

As a second step, the user interactively selects a baseline (Fig. 2B). For fluorescent polysome profiling, the baseline for the fluorescence profile has to be set separately. The baseline is subtracted from the profile values for all further steps. Profiles may also be shifted against each other along the x-axis. Therefore, the user additionally needs to choose a position for alignment on the x-axis, the so-called “x-anchor” (Fig. 2B). The profiles in Fig. 2C, for example, were all aligned along the valley between the monosome and the disome peak, so that the x-value of this position is identical for all profiles. Precise selection of an x-anchor is optionally supported by functions for the automatic detection of local minima, maxima or inflection points. For quantification of profile subsections, the user interactively selects the borders of the area of interest (Fig. 2D), which may also be guided by the functions for automatic detection of local minima, maxima or inflection points. The area under the profile is then approximated by the sum of all data points from the selected start to the selected end position after subtraction of the baseline.

Accurate quantification of individual peaks in polysome profiles can be challenging when neighboring peaks overlap, leading to ambiguous signal contributions. Deconvolution of mammalian polysome profiles is often only feasible in a very restricted range of a profile where peaks are well resolved and can be approximated by a Gaussian distribution. At the beginning and the end of the profile, however, peaks often deviate from symmetry, and small unresolved peaks such as half-mers can distort the shape of larger peaks (Fig. 1D). To address these constraints, QuAPPro allows user-guided deconvolution within a restricted region of a profile. Putative peak positions are automatically identified from the negative local minima of the smoothed second derivative of the profile (Fig. S3A) [18], which is represented by the second-order difference between the individual data values after baseline subtraction and smoothing. The degree of smoothing and the resolution for identifying local minima can be adjusted by the user (Fig. S3B–C). The user then defines the portion of the UV profile to be modeled (Fig. 2E). Non-linear regression with the R function nls is used to estimate the position, standard deviation and height of Gaussian distributions as a model for individual peaks. The positions are restricted to the location of the identified second derivative minima with a tolerance of 1% of the entire profile length to the left and to the right.

All quantification results can be summarized and visualized as a bar plot (or a grouped bar plot, if applicable) after the user assigned conditions to the individual profiles in an interactive table. Absolute or relative values can be displayed. For example, the proportion of polysomes relative to the total area is often measured as a proxy for global translation efficiency. In this case, the user can choose to normalize the polysomal area to the total area (Fig. 2F). Profile quantification can also be used to modify the appearance of the plotted alignment by compensating for discrepancies in the total area or length of the profiles. The amount of cellular material on the gradient can differ due to technical variation or experimental conditions. In addition, sensitivity settings of the UV detector and elution speed may vary between runs, and when profiles recorded with different instruments should be compared, the resolution of the profiles (i.e. the number of data points per time) will not be identical. To address this challenge, QuAPPro offers the option to adjust the areas quantified as “Total” by the user to the largest or longest total area as a reference (Fig. 2C–D, Fig. S4).

## Results and discussion

### Analysis of polysome profiles in LPS-treated macrophages

As an example, we analyzed polysome profiles recorded from RAW264.7 macrophages upon exposure to lipopolysaccharide (LPS; Table S2), which induces a strong response and the production of numerous inflammatory mediators. After loading the PeakTrak files (.pks) into the app (Fig. 2A), we selected the UV absorbance at the end of the profile as a baseline, where the gradient has been eluted completely, but sucrose solution is still passing the detector (Fig. 2B). For a precise selection of the valley between the 80S and the disome peak as x-anchor for alignment, we used the automatic identification of the local minimum that is closest to the click into the plot. The alignment of profiles recorded from the three different conditions indicates a marked decrease of polysomes after 16 h of LPS treatment (Fig. 2C). This is in line with the phosphorylation of the translation initiation factor eIF2α observed in RAW264.7 cells upon prolonged exposure to LPS [19]. To quantify this effect, we selected the region from the 40S peak to the end of the profile as total area, and the region from the disomes to the end of the profile as polysomal area (Figs. 1B and 2D). After performing the quantification of polysomal and total profile areas for three biological replicates, we displayed the means and standard deviations of the polysomal area relative to the total area as a bar plot (Fig. 2F). In addition to the drop after 16 h LPS treatment, the quantification revealed a small increase in the proportion of polysomes after 1 h. This slight effect becomes only apparent in the alignment after adjusting all total areas to the same value (Fig. 2C, Fig. S4). In addition to the decrease in polysomes, the profiles indicate that there is a relative increase especially in the 60S peak. Therefore, we performed deconvolution of the region around the 60S peak, starting from the lower inflection point of the 40S peak to the upper inflection point of the 80S peak (Fig. 2E), and visualized the result as a bar plot showing the 60S peak areas relative to the total area (Fig. S5). Peak deconvolution is also possible with Origin (OriginLab), a commercial general-purpose data analysis and graphing software. We compared deconvolution results obtained in QuAPPro and Origin for the same region after subtracting the same baseline value and obtained nearly identical results (Fig. S6).

### Comparison of QuAPPro to other tools for polysome profile analysis

Some devices for recording UV absorbance offer software with additional options for alignment or quantification of the profiles. The TRIAX Flow Cell software, for example, offers the possibility to align profiles along selected peaks and to adjust the baseline. In conjunction with the siFractor (siTOOLs Biotech) as an adaptor, ÄKTA liquid chromatography systems can be used for polysome profiling. This opens the possibility to use the Unicorn software for overlaying profiles and quantifying areas under sections of the profile. In addition, most labs that routinely perform polysome profiling have established a way to align and quantify profiles with spreadsheet interface programs like Excel, or with custom scripts in R/Python. Shaffer and Rollins provide an R script with their protocol for fluorescent polysome profiling, which only allows interactive quantification of areas under UV and fluorescence profiles in parallel [7]. Several alternative tools are available on GitHub but offer more limited functionality than QuAPPro. PolyPlotMat (https://github.com/NoahLind01/PolyPlotMat) is a MATLAB-based application for visualization, comparison, and quantification of UV absorbance and fluorescence polysome profiles, including support for multiple fluorescence channels. However, it requires a MATLAB installation, does not support peak deconvolution, and only accepts CSV input files without documentation on software compatibility. The R package polysome.profileR (https://github.com/jfavate/polysome.profileR) is designed for plotting BioComp gradient profiles. It does not support fluorescence signals, interactive exploration, or quantitative analysis, and lacks documentation. The Interactive Polysome Profile Analyser (IPPA, https://github.com/ChiaraGiacomelli/IPPA) is a basic shiny application for plotting and area-under-the-curve calculations of single UV polysome profiles. It does not allow alignment of multiple profiles, peak deconvolution or analysis of fluorescence signals. Taken together, existing tools address individual aspects of polysome profile analysis, whereas QuAPPro provides an integrated and accessible solution for interactive alignment and quantitative comparison across profiles.

## Limitations

Although QuAPPro is very flexible concerning the format of input files, there are some limitations. Every run has to be imported as a separate file, because only one column can be indicated per UV or fluorescence profile. For the same reason, only one fluorescence channel can be analyzed in parallel to the UV profile. UV and fluorescence profiles need to be stored as two columns with corresponding measurements in the same row. When UV and fluorescence profiles are concatenated in the same column, the fluorescence profile cannot be processed.

By normalizing the total area and length of aligned profiles, differences in elution time or temporal resolution between devices can be adjusted for. Variations in sucrose density, however, may still distort the profiles. Li et al. implemented correlation-optimized warping in MATLAB to align and analyze large numbers of polysome profiles for investigating ribosome biogenesis in yeast [5]. In contrast, QuAPPro can only stretch or shrink a profile by a given factor.

Quantification results can be summarized and visualized as bar plots showing means and standard deviations of replicates or groups, but statistical comparisons are not possible with QuAPPro. Also the analysis of advanced approaches like the measurement of ribosome elongation speed with harringtonine run-offs may require further downstream processing that is not included in the app. For this purpose, quantification results can be downloaded as CSV files.

## Conclusions

QuAPPro is currently the only well-documented application that combines polysome profile plotting, interactive alignment, and quantitative analysis within a single analysis suite. It is also the only tool that is hosted online and therefore does not require any local software installation or programming skills. The app allows users to import many different input file formats, so that it is not limited to data of a specific device or software. Due to the functions for normalizing the total area and length of the profile, even profiles recorded with different devices can be aligned and analyzed together. It is interactive and therefore highly flexible, as users can decide along which peaks or valleys they would like to align or quantify their profiles. Furthermore, QuAPPro offers automatic detection of local minima, maxima, and inflection points, which renders the analysis very efficient, accurate and reproducible. Not only the results of a quantification or alignment can be downloaded. Also, an entire analysis can be exported and re-imported, so that every step of the analysis is well documented. In addition, all plots displayed in QuAPPro can be downloaded as PDF files at any point of the analysis. As a showcase, the graphs presented in Fig. 2 were retrieved from QuAPPro without any further modifications. Therefore, the app does not only provide a fast, robust, and interactive way to quantify polysome profiles, but also generates ready-to-publish plots.

### Availability and requirements

QuAPPro can be accessed independent of the platform as a web-based shiny application at https://www.umm.uni-heidelberg.de/mi3/biochemie/tools/quappro. For running QuAPPro locally, the R code can be downloaded from our GitHub project repository at https://github.com/johannaschott/QuAPPro. QuAPPro was written in R v4.0.5. In addition, the R packages shiny [17], shinythemes [20], colourpicker [21], stringr [22], colorspace [23], shinyalert [24], shinybusy [25], DT [26], ggplot2 [27], ggbeeswarm [28], dplyr [29] and cicerone [30] are used. QuAPPro is available under the GPL v3.0 license, and there is no restriction to use by non-academics.

## Supplementary Information

Below is the link to the electronic supplementary material.

Supplementary Material 1

## Data Availability

All polysome profiles presented in this manuscript are available at our GitHub repository at https://github.com/johannaschott/QuAPPro/tree/main/profile_data. Table S2 lists the experimental conditions and the devices that were used for recording the profiles.

## Abbreviations

RQC: Ribosome quality control
Har: Harringtonine
ANI: Anisomycin
LPS: Lipopolysaccharide
RPF: Ribosome protected fragment

## References

[1] Warner JR, Knopf PM, Rich A. A multiple ribosomal structure in protein synthesis. Proc Natl Acad Sci U S A. 1963;49(1):122–9. 13998950 10.1073/pnas.49.1.122 PMC300639

[2] Wettstein FO, Staehelin T, Noll H. Ribosomal aggregate engaged in protein synthesis: characterization of the ergosome. Nature. 1963;197:430–5. 14000153 10.1038/197430a0

[3] Eshraghi M, Karunadharma PP, Blin J, Shahani N, Ricci EP, Michel A, et al. Mutant Huntingtin stalls ribosomes and represses protein synthesis in a cellular model of huntington disease. Nat Commun. 2021;12(1):1461. 33674575 10.1038/s41467-021-21637-y PMC7935949

[4] Popper B, Burkle M, Ciccopiedi G, Marchioretto M, Forne I, Imhof A, et al. Ribosome inactivation regulates translation elongation in neurons. J Biol Chem. 2024;300(2):105648. 38219816 10.1016/j.jbc.2024.105648 PMC10869266

[5] Li Z, Lee I, Moradi E, Hung NJ, Johnson AW, Marcotte EM. Rational extension of the ribosome biogenesis pathway using network-guided genetics. PLoS Biol. 2009;7(10):e1000213. 19806183 10.1371/journal.pbio.1000213 PMC2749941

[6] Lehmann JA, Lindner D, Sung HM, Stoecklin G. E3 ubiquitin ligase RNF10 promotes dissociation of stalled ribosomes and responds to ribosomal subunit imbalance. Nat Commun. 2024;15(1):10350. 39609413 10.1038/s41467-024-54411-x PMC11604940

[7] Shaffer D, Rollins JA. Fluorescent polysome profiling in caenorhabditis elegans. Bio Protoc. 2020;10(17):e3742. 33659402 10.21769/BioProtoc.3742 PMC7842706

[8] Doring K, Ahmed N, Riemer T, Suresh HG, Vainshtein Y, Habich M, et al. Profiling Ssb-Nascent chain interactions reveals principles of Hsp70-Assisted folding. Cell. 2017;170(2):298–311. e20. 28708998 10.1016/j.cell.2017.06.038 PMC7343536

[9] Ingolia NT, Ghaemmaghami S, Newman JR, Weissman JS. Genome-wide analysis in vivo of translation with nucleotide resolution using ribosome profiling. Science. 2009;324(5924):218–23. 19213877 10.1126/science.1168978 PMC2746483

[10] Cenik C, Cenik ES, Byeon GW, Grubert F, Candille SI, Spacek D, et al. Integrative analysis of RNA, translation, and protein levels reveals distinct regulatory variation across humans. Genome Res. 2015;25(11):1610–21. 26297486 10.1101/gr.193342.115 PMC4617958

[11] Ferguson L, Upton HE, Pimentel SC, Mok A, Lareau LF, Collins K, et al. Streamlined and sensitive mono- and di-ribosome profiling in yeast and human cells. Nat Methods. 2023;20(11):1704–15. 37783882 10.1038/s41592-023-02028-1 PMC11276118

[12] Bertolini M, Fenzl K, Kats I, Wruck F, Tippmann F, Schmitt J, et al. Interactions between nascent proteins translated by adjacent ribosomes drive homomer assembly. Science. 2021;371(6524):57–64. 33384371 10.1126/science.abc7151 PMC7613021

[13] Kopeina GS, Afonina ZA, Gromova KV, Shirokov VA, Vasiliev VD, Spirin AS. Step-wise formation of eukaryotic double-row polyribosomes and circular translation of polysomal mRNA. Nucleic Acids Res. 2008;36(8):2476–88. 18310103 10.1093/nar/gkm1177 PMC2377419

[14] Afonina ZA, Myasnikov AG, Shirokov VA, Klaholz BP, Spirin AS. Conformation transitions of eukaryotic polyribosomes during multi-round translation. Nucleic Acids Res. 2015;43(1):618–28. 25520190 10.1093/nar/gku1270 PMC4288168

[15] Yoshikawa H, Larance M, Harney DJ, Sundaramoorthy R, Ly T, Owen-Hughes T et al. Efficient analysis of mammalian polysomes in cells and tissues using Ribo Mega-SEC. Elife. 2018;7:e36530. 30095066 10.7554/eLife.36530 PMC6086667

[16] Team RC. R: A language and environment for statistical computing. 2024.

[17] Chang W, Cheng J, Allaire J, Sievert C, Schloerke B, Xie Y et al. Shiny: web application framework for R. 2023.

[18] Lu J, Trnka MJ, Roh SH, Robinson PJ, Shiau C, Fujimori DG, et al. Improved peak detection and deconvolution of native electrospray mass spectra from large protein complexes. J Am Soc Mass Spectrom. 2015;26(12):2141–51. 26323614 10.1007/s13361-015-1235-6 PMC5067139

[19] Kimura K, Ito S, Nagino M, Isobe K. Inhibition of reactive oxygen species down-regulates protein synthesis in RAW 264.7. Biochem Biophys Res Commun. 2008;372(1):272–5. 18486599 10.1016/j.bbrc.2008.05.036

[20] Chang W. Shinythemes: themes for shiny. 2021.

[21] Attali D. Colourpicker: a colour picker tool for shiny and for selecting colours in plots. 2023.

[22] Wickham H. Stringr: simple, consistent wrappers for common string operations. 2023.

[23] Zeileis A, Hornik K, Murrell P. Escaping rgbland: selecting colors for statistical graphics. Comput Stat Data an. 2009;53(9):3259–70.

[24] Attali D, Edwards T, shinyalert. Easily create pretty popup messages (Modals) in ‘shiny’. 2024.

[25] Meyer F, Perrier V. Shinybusy: busy indicators and notifications for ‘shiny’ applications. 2024.

[26] Xie X, Cheng J, Tan X. DT: A wrapper of the JavaScript library ‘DataTables’. 2024.

[27] Wickham H. ggplot2: elegant graphics for data analysis. New York: Springer-; 2016.

[28] Clarke E, Sherrill-Mix S, Dawson C. ggbeeswarm: Categorical scatter (Violin Point) Plots. 2023.

[29] Wickham H, François R, Henry L, Müller K, Vaughan D. dplyr: A grammar of data manipulation. 2023.

[30] Coene J. Cicerone: provide tours of ‘shiny’ applications. 2021.

