# Supplementary material for: QuAPPro: an R shiny app for quantification and alignment of polysome profiles
Source: BMC Bioinformatics. 2026 Jan 22;27:22. doi: 10.1186/s12859-026-06379-2 (PMC12849056; doi:10.1186/s12859-026-06379-2)
Supplement: Supplementary file 1 — Supplementary Material 1 [file 12859_2026_6379_MOESM1_ESM.docx]

**Supplement**


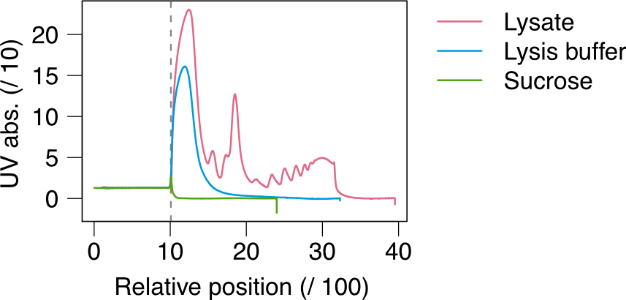


**Figure S1.** **Baseline of polysome profiles.** UV absorbance was recorded with a Teledyne Isco Foxy Jr. from a sucrose-density gradient alone (green), a gradient loaded with lysis buffer (blue) or with a lysate of HEK293 cells (red).

**
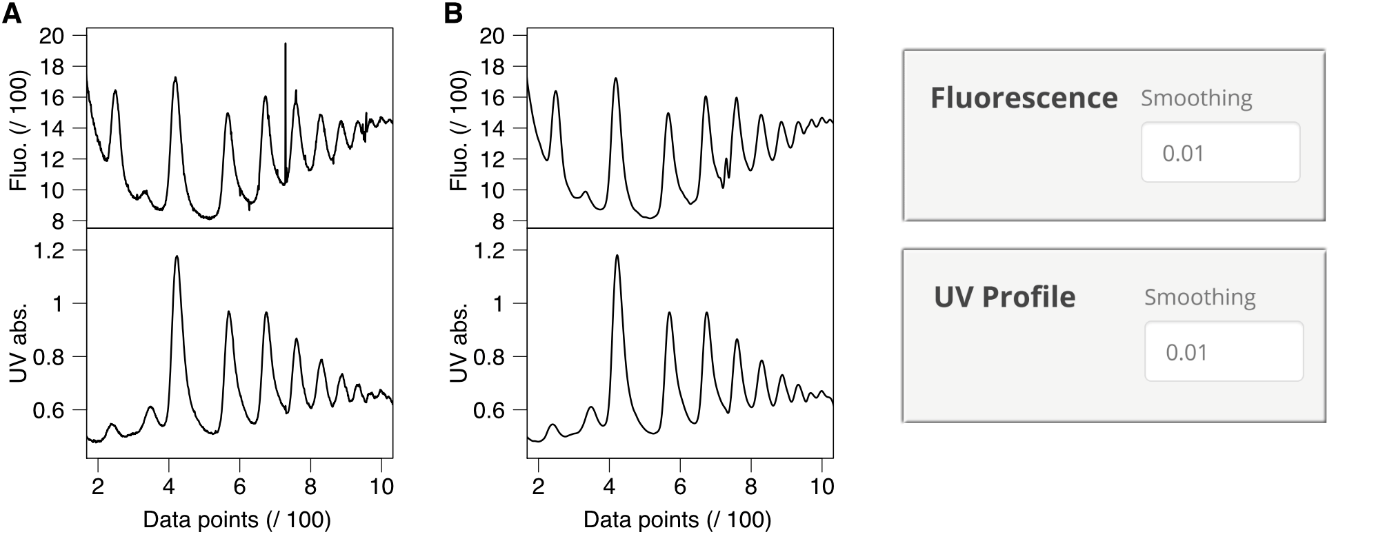
**

**Figure S2. Smoothing of UV and fluorescence profiles. A.** Unsmoothed fluorescence and UV absorbance profile of yeast cells expressing GFP-tagged Ssb protein. **B.** Profiles shown in A. after smoothing with a smoothing parameter of 0.01.

**
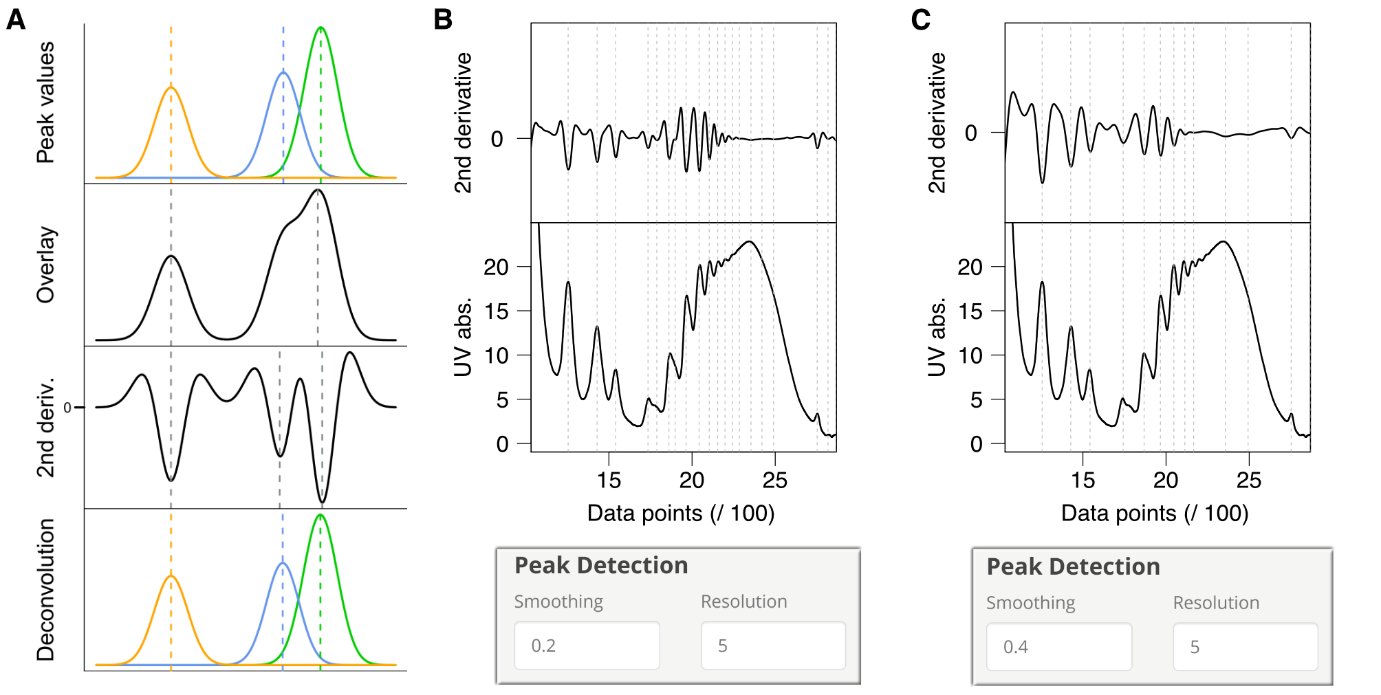
**

**Figure S3. Identification of peak positions using negative second derivative minima. A.** Three partially overlapping Gaussian peaks (Peak values) result in a profile where the middle peak does not produce a local maximum (Overlay). The negative minima of the second derivative (2nd deriv.) identify peak positions, which are used for resolving individual peaks (Deconvolution). **B.** Identification of putative peak positions (dashed grey lines) from a polysome profile in QuAPPro with default parameters. **C.** Identification of putative peaks (dashed grey lines) with increased smoothing of the second derivative.


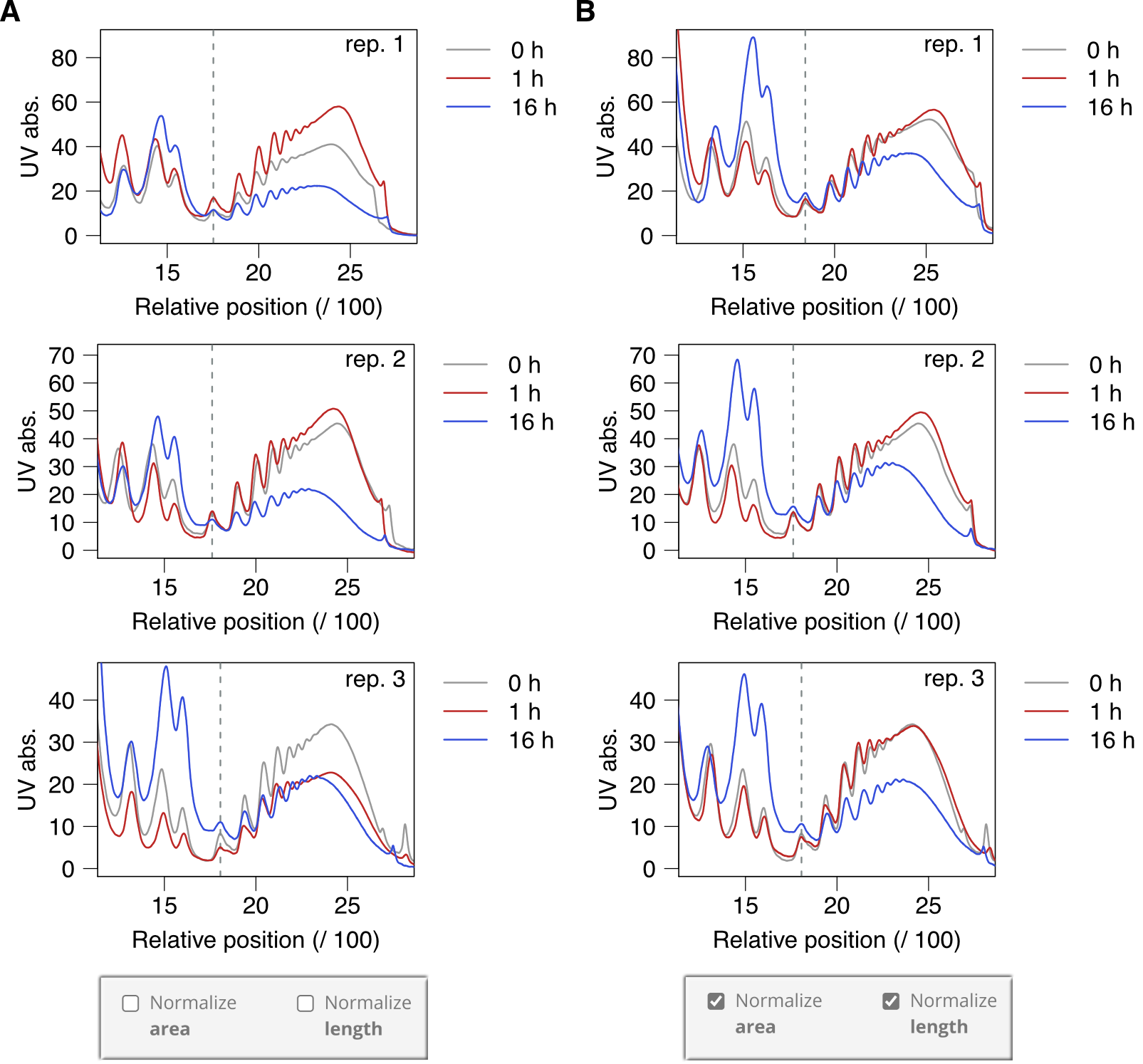


**Figure S4. Normalization of polysome profiles to the total area and length. A.** Polysome profiles of RAW264.7 cells after 0 h, 1 h and 16 h of LPS treatment, as used for quantification in Fig. 2F. **B.** Polysome profiles as in A. after normalization to the total area and length, so that the small but systematic relative increase in polysomes after 1 h of LPS treatment is visible.


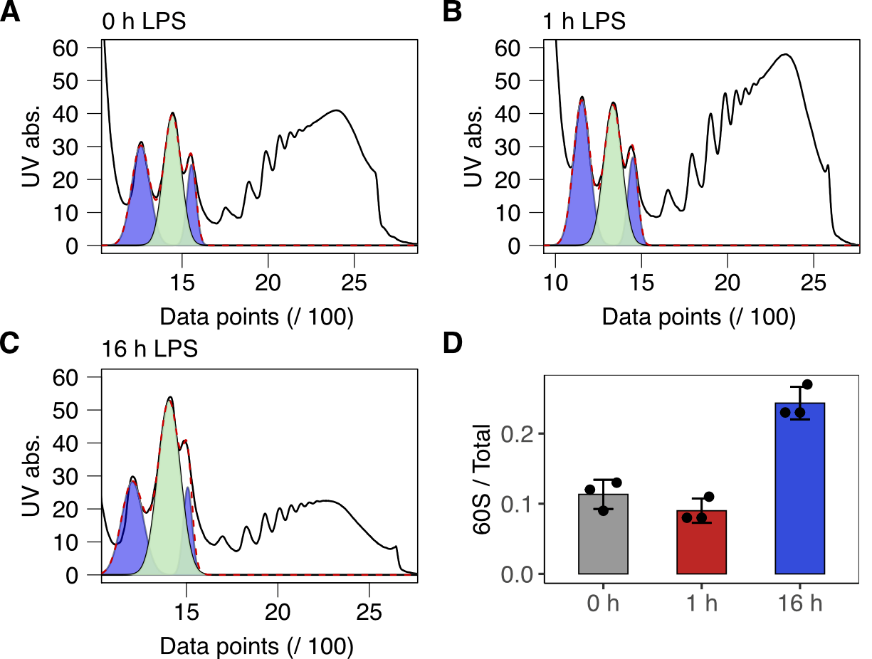


**Figure S5. Peak deconvolution and quantification of the relative 60S area. A.** Deconvolution of the region from the 40S to the 80S peak for quantification of the 60S peak (green) in a polysome profile of RAW264.7 cells. **B.** As in A., after 1 h LPS treatment. **C.** As in A., after 16 h LPS treatment. **D.** Bar plot of the 60S peak area relative to the total profile area as quantified from three biological replicates.


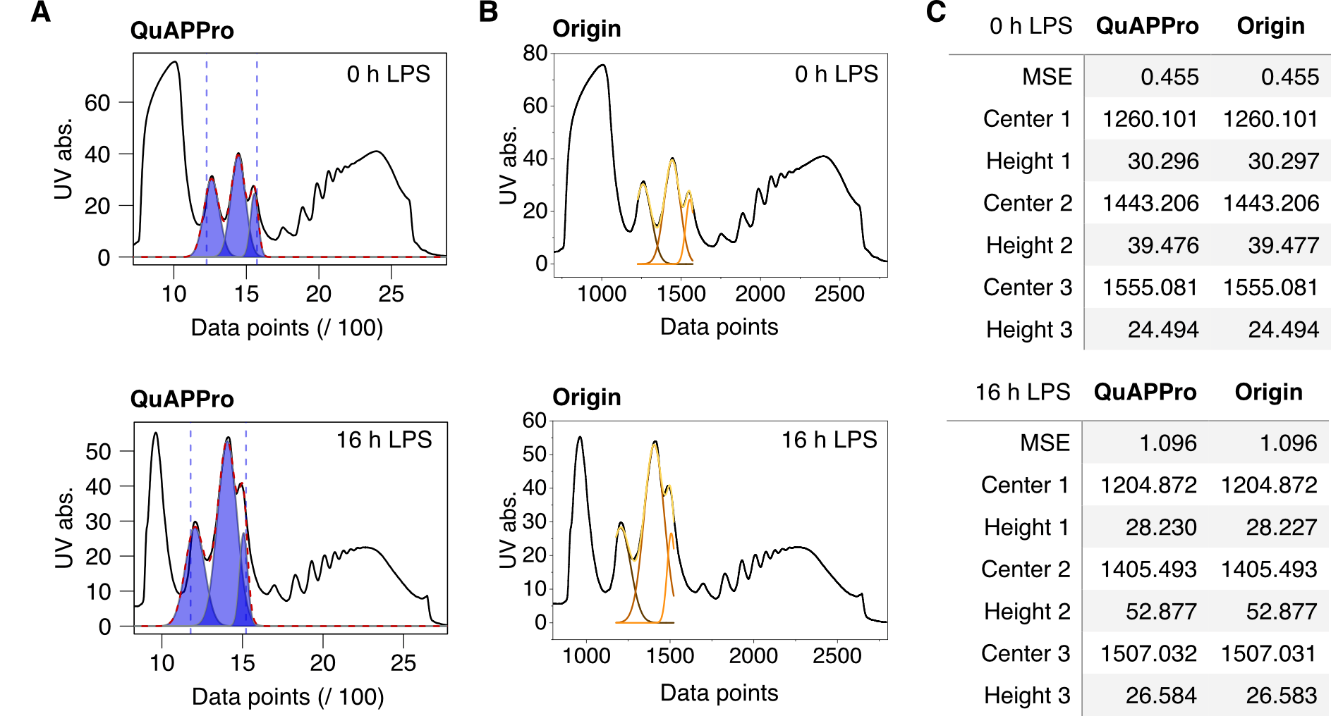


**Figure S6. Comparison of peak deconvolution results with QuAPPro und Origin. A.** Deconvolution of the 40S, 60S and 80S peaks in polysome profiles of RAW264.7 cells after 0 h and 16 h of LPS with QuAPPro. Peak models are based on the region between the two vertical dashed lines, which were identified as inflection points. **B.** Deconvolution of the 40S, 60S and 80S peaks in the same region as in A. with the commercial software Origin. The same baseline value was subtracted from the profile as in A., and zero was set as a fixed base value for all peaks. **C.** Mean squared error (MSE) of the peak model, the center positions and the heights of the three analyzed peaks as obtained with QuAPPro and Origin.

| **File type** | **Skip lines** | **Column delimiter** | **Decimal separator** | **UV column** | **Fluorescence column** |
| --- | --- | --- | --- | --- | --- |
| PeakTrak | 0 | space | comma | 3 | NA |
| TRIAX (UV) | 47 | comma | point | 4 | NA |
| TRIAX (+ Fl.) | 51 | comma | point | 5 | 3 |
| PrimeView | 2 | space | comma | 2 | NA |

**Table S1: Default import parameters of the predefined file formats.** This table lists import settings for four common file types exported from the PeakTrak, TRIAX and PrimeView software. The decimal separator (and for CSV files also the column delimiter) may depend on the language settings of the operating system, and might have to be adjusted by the user. Some file types contain a header with additional information about the run, which has to be skipped for import. The number of lines to be skipped may differ between software versions or due to user settings.

| Cell line | | | | | |
| --- | --- | --- | --- | --- | --- |
| RAW264.7 | | Fig. 1A - C, 1F, 2, S3 - S6 | | | |
| HeLa | | Fig. 1D | | | |
| Yeast BY4741 Ssb1-GFP | | Fig. 1E, S2 | | | |
| HEK293 | | Fig. S1 | | | |
| Treatment | | | | | |
| 100 ng/ml LPS (E. coli O111:B4, Sigma L2630) | | | Fig. 1A - B, 2, S4 - S6 | | |
| 2 µg/ml HT (Bertin Pharma CAY-15361) for the indicated time periods at RT before addition of 100 µg/ml CHX | | | Fig. 1C | | |
| 0.2 µg/ml Anisomycin | | | Fig. 1D | | |
| Limited digest of the lysate with 300 U RNase T1 per 7 A260 Units and 500 ng RNase A per 7 A260 Units for 45 min at room temperature | | | Fig. 1F | | |
| Lysis procedure | | | | | |
| Mammalian cells were lysed with the indicated lysis buffer in the presence of cycloheximide (CHX). After tumbling the lysates for 10 min at 4°C, nuclei and cell debris were removed by centrifugation at 9,300 × g for 10 min at 4°C. | | | | Fig. 1A - D, 1F, 2, S1, S3 - S6 | |
| Growing yeast cultures (OD600 of 0.5-0.6 in YPD) were filtered and lysed by mixer milling (2 min, 30 Hz, MM400 Retsch) with liquid nitrogen in lysis buffer. The frozen lysate powder was stepwise added to 100 μl hexokinase buffer (20 mM Tris-HCl pH 8.0, 40 mM NaPO_4_ pH 7.0, 0.2% glucose) containing 100 U of hexokinase in a 10 ml beaker while stirring with a magnetic flea to immediately deplete the ATP. After everything was thawed, the lysates were transferred to Eppendorf tubes and spun for 2 min at 30,000 g and 4°C to remove nuclei and cell debris. | | | | Fig. 1E,  S2 | |
| Lysis buffer | | | | | |
| 15 mM Tris HCl (pH 7.4), 15 mM MgCl_2_, 300 mM NaCl, 100 µg/ml CHX, 1% Triton-X-100, 0.1% β-mercaptoethanol, 200 U/ml RNAsin (Promega), 1 complete Mini Protease Inhibitor Tablet (Roche) per 10 ml | | | Fig. 1A - B, 2, S3 - S6 | | |
| 20 mM Tris HCl (pH 7.5), 150 mM NaCl, 5 mM MgCl_2_, 1 mM DTT, 100 µg/ml CHX, 1% Triton X-100, 1 complete Mini Protease Inhibitor Tablet per 10 ml | | | Fig. 1C - D, 1F | | |
| 20 mM HEPES pH 8.0, 140 mM KCl, 10 mM MgCl_2_, 0.1% NP-40, 100 µg/ml CHX, 1 mM PMSF, 2 × protease inhibitors (Complete EDTA-free, Roche), 0.02 U/μl DNaseI (recombinant DNaseI, Roche), 20 μg/mL leupeptin, 20 μg/mL aprotinin, 1 μg/uL E-64, 40 μg/mL bestatin | | | Fig. 1E, S2 | | |
| 20 mM Tris-HCl (pH 7.4), 10 mM MgCl_2_, 200 mM KCl_2_, 1% NP-40, 100 µg/ml CHX, 2 mM DTT, 200 U/ml RNAsin, 1 complete Mini Protease Inhibitor Tablet per 10 ml | | | Fig. S1 | | |
| Sucrose-density gradients | | | | | |
| 17.5% - 50% (w/v) sucrose (in 15 mM Tris HCl at pH 7.4, 15 mM MgCl_2_, 300 mM NaCl) | | | Fig. 1A - B, 2, S3 - S6 | | |
| 17.5% - 50% (w/v) sucrose (in 20 mM Tris HCl pH 7.5, 150 mM NaCl, 5 mM MgCl_2_) | | | | | Fig. 1C - D, 1F |
| 5% - 45% (w/v) sucrose gradients (in 20 mM HEPES pH 8.0, 140 mM KCl, 10 mM MgCl_2_, 0.1 mg/ml CHX) | | | | | Fig. 1E, S2 |
| 17.5% - 50% (w/v) sucrose (in 20 mM Tris-HCl at pH 7.4, 10 mM MgCl_2_, 200 mM KCl) | | | | | Fig. S1 |
| Centrifugation settings | | | | | |
| 2.5 h, 35,000 rpm at 4°C in a SW60 Ti rotor | Fig. 1A - B, 1F, 2, S3 - S6 | | | | |
| 2 h, 40,000 rpm at 4°C in a SW60 Ti rotor | Fig. 1C - D, S1 | | | | |
| 2.5 h, 35,000 rpm at 4°C in a SW40 rotor | Fig. 1E, S2 | | | | |
| Device and software | | | | | |
| Teledyne Isco Foxy Jr., PeakTrak software | Fig. 1A - D, 1F, 2, S1, S3 - S6 | | | | |
| Piston Gradient Fractionator and TRIAX detector (FC-2 dual wavelength flow cell, BioComp), FlowCell software | Fig. 1E, S2 | | | | |

**Table S2: Experimental and technical conditions of polysome profiles.** This table lists cell lines, treatment conditions, lysis conditions, centrifugation settings and information about the devices used for recording the profiles.
